# Size matters at deep-sea hydrothermal vents: different diversity and habitat fidelity patterns of meio- and macrofauna

**DOI:** 10.3354/meps11078

**Published:** 2015-02-03

**Authors:** Sabine Gollner, Breea Govenar, Charles R. Fisher, Monika Bright

**Affiliations:** 1 Department of Marine Biology, University of Vienna, Althanstrasse 14, 1090 Vienna, Austria; 2 German Center for Marine Biodiversity Research (DZMB), Senckenberg am Meer, Am Südstrand 44, 26382 Wilhelmshaven, Germany; 3 Royal Netherlands Institute for Sea Research (NIOZ), Ecosystem Studies, Korringaweg 7, 4401 NT Yerseke, The Netherlands; 4 Biology Department, Rhode Island College, 600 Mt. Pleasant Ave., Providence, Rhode Island 02908, USA; 5 Department of Biology, 208 Mueller Laboratory, The Pennsylvania State University, University Park, Pennsylvania 16802, USA

**Keywords:** Body size, Diversity, Habitat fidelity, Stress, Meiofauna, Macrofauna, Deep sea, Hydrothermal vent

## Abstract

Species with markedly different sizes interact when sharing the same habitat. Unravelling mechanisms that control diversity thus requires consideration of a range of size classes. We compared patterns of diversity and community structure for meio- and macrofaunal communities sampled along a gradient of environmental stress at deep-sea hydrothermal vents on the East Pacific Rise (9° 50′ N) and neighboring basalt habitats. Both meio- and macrofaunal species richnesses were lowest in the high-stress vent habitat, but macrofaunal richness was highest among intermediate-stress vent habitats. Meiofaunal species richness was negatively correlated with stress, and highest on the basalt. In these deep-sea basalt habitats surrounding hydrothermal vents, meiofaunal species richness was consistently higher than that of macrofauna. Consideration of the physiological capabilities and life history traits of different-sized animals suggests that different patterns of diversity may be caused by different capabilities to deal with environmental stress in the 2 size classes. In contrast to meiofauna, adaptations of macrofauna may have evolved to allow them to maintain their physiological homeostasis in a variety of hydrothermal vent habitats and exploit this food-rich deep-sea environment in high abundances. The habitat fidelity patterns also differed: macrofaunal species occurred primarily at vents and were generally restricted to this habitat, but meiofaunal species were distributed more evenly across proximate and distant basalt habitats and were thus not restricted to vent habitats. Over evolutionary time scales these contrasting patterns are likely driven by distinct reproduction strategies and food demands inherent to fauna of different sizes.

## INTRODUCTION

Animals occur in a wide range of sizes, ranging from <100 micrometers in length and a few micrograms in weight up to several meters and thousands of kilograms. For centuries, scientists have investigated the evolution and physiological constraints of size variation among animals. In general, the larger a species, the longer its life span and generation time, and the greater its maintenance energy costs (Schmidt-Nielsen 1984, Woodward et al. 2005, Bonner 2006). In the marine benthic environment, scientists routinely distinguish 2 size groups: the small ‘meiofauna’ and the large ‘macrofauna’ (Giere 2009). Not only the size per se, but different life history traits, such as reproductive rates and modes, intrinsically linked to size, justify the differentiation of animals into these size classes. Permanent meiofauna, such as nematodes or copepods, remain small as adults. They generally produce few offspring, which de velop relatively fast and directly from juveniles into adults. Permanent meiofauna have several generations per year, and most exhibit a mobile lifestyle. In contrast, macrofauna, such as gastropods and polychaetes, generally produce more offspring, which develop from larvae that disperse in the water column and then metamorphose into benthic juveniles that develop into adults. Macrofauna have longer generation times than meiofauna and exhibit a variety of mobile and sessile lifestyles (Warwick 1984, Giere 2009).

The responses of meiofaunal and macrofaunal communities in the marine benthic habitat may differ when exposed to the same environmental conditions. For example, along a gradient in environmental stress, larger animals may have a wider range of adaptations to cope with extreme conditions, including high biomass or thick protective coverings to protect against changes in temperature. However, smaller animals may have other adaptations, such as greater mobility. Mobility and behavioral adaptations allow selection of microhabitat niches, with different realized environmental conditions. Furthermore, because of their differences in size, and dispersal and life history traits, meio- and macrofauna may vary in endemicity patterns at large spatial scales, and habitat utilization may vary at smaller spatial scales. Generalists exhibit a broad realized niche width and evolve in temporally varying heterogeneous environments, whereas specialists have a narrower realized niche and are expected to arise under constant homogeneous environments (Kassen 2002). Furthermore, the relationship between productivity and diversity may vary between the meioand macrofauna, because of the differences in their food demands and relative abundances.

Deep-sea hydrothermal vents are among the most productive ecosystems in the ocean, in contrast to the low productivity that characterizes the surrounding abyssal deep-sea plains (Etter & Mullineaux 2001). Unlike typical shallow-water marine habitats, areas of high productivity at vents coincide with harsh or stressful environmental conditions. At hydrothermal vents, high *in situ* primary chemoautotrophic production is limited to areas of active hydrothermal flow, whereas proximate basalt lacks *in situ* primary production with limited input from proximate vents. Visually dominant megafauna live in symbiosis with chemoautotrophic bacteria and require exposure to chemical energy sources, and serve as a visible indicator of areas with high productivity (Van Dover 2000, Etter & Mullineaux 2001). Environmental stresses associated with hydrothermal vent habitats include high temperatures, high metal concentrations, high concentrations of reduced chemicals, and low oxygen concentrations, as well as extreme variation in all of these parameters over small distances and short time periods (Luther et al. 2001, Von Damm & Lilley 2004, Le Bris et al. 2006, Fisher et al. 2007, Le Bris & Gaill 2007, Bates et al. 2010).

In the Axial Summit Trough (AST) of the East Pacific Rise (EPR), active hydrothermal vents are patchily distributed but densely populated by different types of megafauna (Pompeii worms, tubeworms, and mussels) associated with high productivity and biochemically and physiologically stressful environmental conditions. The dominant megafauna vary ac cording to different ranges in temperature and chemical composition and act as foundation species (Van Dover 2000, Govenar & Fisher 2007). The Pompeii worm habitat, with the highest and most variable temperatures, is the most extreme habitat occupied by metazoans. Animals living in the tubeworm habitat are exposed to a high–intermediate range of hydrothermal stressors, and in the mussel habitat to intermediate–low stressors. Away from hydrothermal flow, the AST lacks these foundation species. Thus, the habitat is generally bare basalt, with lower productivity and less environmental stress, given the absence of vent fluids (see Table 1) (Johnson et al. 1988, Di Meo-Savoie et al. 2004, Le Bris et al. 2006, Le Bris & Gaill 2007).

Distinct communities of macrofauna and meiofauna associate with different characteristic foundation species in each type of vent habitat. Previous studies of vent community structure have focused primarily on either macrofauna (e.g. Van Dover 2003, Govenar et al. 2005, Galkin & Goroslavskaya 2008) or meiofauna (e.g. Zekely et al. 2006, Copley et al. 2007, Gollner et al. 2007) associated with one type of foundation fauna. Other studies have focused on a particular taxonomic group (i.e. gastropods) (Mills et al. 2007b, Matabos et al. 2008) from different environments, and we recently examined meiofaunal diversity along a hydrothermal fluid gradient at the 9° 50′ N EPR (Gollner et al. 2010). However, no previous study has compared macro- and meiofaunal communities across the range of vent habitats.

In this study, we test the hypothesis that the meioand macrofauna components of a community, which exhibit physiological capabilities and life history traits inherent to their size (Powell 1989, Brown et al. 2004), respond differently to the same environmental conditions. Here we combine ana lyses of meio- and macrofaunal communities, sampled from the same habitats and often the same collections, from hydrothermal vents and proximate basalt within the AST of the EPR (9° 50′ N) to explore *in situ* diversity of meio- and macrofaunal communities along a stress gradient. In addition, we incorporate samples collected from ~1 km outside the AST (referred to as vent-distant) to evaluate distribution and thus specialization patterns of vent meio- and macrofauna. Although most vent macrofauna are considered to be restricted to vent habitats (Tunnicliffe 1992), whether meiofauna previously found only at vents and in close vicinity to vents in the AST are also restricted to this habitat remains an unanswered question. Through comparative analyses, we also examined the effect of body size on community diversity and relative abundance of specialists and generalists in habitats characterized by opposing environmental stress and productivity levels.

## MATERIALS AND METHODS

### Study sites and collections

All samples were collected using the deep submergence vehicle (DSV) ‘Alvin’ in the 9° 50′ N region of the EPR at 2500 m depth. In order to cover the full natural hydrothermal stress gradient, we analyzed samples from (1) Pompeii worm, (2) tubeworm, (3) mussel, and (4) proximate basalt collections (Table 1). Because the samples were from different habitats, with different substrates (basalt and sulfide chimneys) and types of foundation species, our sampling required multiple types of sampling devices. All details on *in situ* sampling methods, as well as information on exact longitude and latitude and a geographic map of the region can be found in Van Dover (2003), Govenar et al. (2005) and Gollner et al. (2010).

Temperature was measured prior to all collections using the temperature probes of DSV ‘Alvin’. Chemical measurements were obtained at the same time and sites as faunal collections from tubeworm and mussel communities (meiofauna collection) and basalt habitats (see Table 1, Le Bris et al. 2006). For the sites inhabited by the Pompeii worm, we present chemical measurements from a range of similar habitats in the region (Table 1). The chemical measurements are used to broadly define the hydrothermal stress gradient, rather than characterizing the exact environment experienced by the organisms. Faunal collections were obtained from several sites within the 9° 50′ N EPR area, and some of the data presented here were published previously (see Table 1). The new species abundance data are presented in Tables S1 & S2 in the Supplement at www.int-res.com/articles/suppl/m520p057_supp.pdf. Meio- and macrofauna were from the same collections, with the lone exception of meiofauna and macrofauna components of the mussel bed communities (which were nonetheless taken from the same East Wall site). Macrofaunal communities vary little among mussel beds in this area of the EPR (Van Dover 2003), and for this study we therefore used mussel bed macrofauna data from Van Dover (2003) (samples from East Wall P1-P6).

The vent-distant basalt sample outside the AST (vent-distant B1) was collected at 9° 50.41′ N, 104° 17.57′ W, ~900 m away from the AST site Tica (9° 50.41′ N, 104° 17.50′ W) during Alvin dive 4264 in 2006. During the same dive, 2 sediment samples (sediments accumulated in basalt cracks: vent-distant S1 and S2) were collected at 9° 50.41′ N, 104° 18.11′ W, ~1.1 km away from the AST site Tica. This area outside the AST is characterized by lava pillows partly covered with small amounts of sediment that have accumulated in cracks in the basalt. The large basalt sample was collected with the submersible’s mechanical arm and placed into an isolated and sealed box on the basket of Alvin. The 2 sediment samples were collected by suction with the pelagic pumps (lined with a 32 μm net) from the submersible.

### Sample processing and data analyses

After sampling, fauna were separated into meioand macrofauna, fixed in 4% buffered formaldehyde and transferred to 70% ethanol. The meiofauna component passed through a 1 mm net and was collected on 32 or 63 μm sieves (Giere 2009; see Gollner et al. 2010 for details). No upper size limit was chosen for the macrofauna, and the lower size limit was chosen at 32 or 63 μm to avoid missing of potential macrofauna species (Gage et al. 2002). We consider macrofauna >1 mm as adult, and macrofauna in the fraction from 32 μm to 1 mm as juvenile. We acknowledge that not all macrofaunal specimens >1 mm were necessarily mature, but macrofaunal specimens in our samples <1 mm were indeed juveniles (Mills et al. 2007a, Tyler et al. 2008). For the mussel macrofauna data, the lower size limit was 263 μm (Van Dover 2003). We note that most juvenile macrofauna from vents (their smallest reported form) exceed 263 μm (Mills et al. 2007a), and we found no additional macrofauna species at the Pompeii worm and tubeworm communities in the 32 to 1000 μm size class; thus we infer that the different sieve size used for mussel macrofauna had no influence on species richness. All macrofauna (>105 000 ind.) were identified to species. All meiofauna (>69 000 ind.) were counted and classified into higher taxa (i.e. nematodes, copepods, ostracods, acari). All, or at least 300 ind. per higher taxon in each collection, were then identified to species or to a putative species. Specimens were identified to the lowest possible taxon (i.e. genus) and were further discriminated within our samples to a putative species. This procedure was chosen for rare putative species (e.g. only one individual, one sex) belonging to complex genera. Cumulative species-effort curves for each higher taxon showed that sampling effort was sufficient to describe the communities (for details see Gollner et al. 2007, 2010).

Macrofaunal data from tubeworm (Govenar et al. 2005) and mussel (Van Dover 2003) habitats were recalculated to species abundances per 10 cm^2^ sea floor area for comparison to the meiofauna data. The foundation species *Alvinella caudata*, *A. pompejana*, *Bathymodiolus thermophilus* (and its kleptoparasitic symbiont *Branchipolynoe symmytilida)*, *Tevnia jerichonana*, *Oasisia alvinae*, and *Riftia pachyptila* were excluded from analyses because they provide habitat for other fauna and do not directly compete with either the meiofauna or other macrofauna. All macrofauna taxa <1 mm were juveniles (i.e. displaying a juvenile form — e.g. polychaetes with just a few chaetigers, or gastropod larvae) and often could be identified to species level following the identification key of Mills et al. (2007a). Juvenile macrofauna, which could not be identified to species level (e.g. some gastropod larvae), were included in abundance calculations but excluded from diversity calculations.

Gained sample coverage calculated using iNEXT (Chao & Jost 2012) indicated high sample coverage (mean >95%) for the meio- and the macrofauna in all samples, supporting the efficacy of our sampling strategy (Table 2). We are aware that varying sampling size can influence species richness and we compensate for this potential bias using sized-based and coverage-based richness calculations (see below). That we obtained our samples in different years could have influenced species richness as well; however, we assume this factor has very little influence. In this region, volcanic eruptions occur with a frequency of about 15 yr (Shank et al. 1998, Tolstoy et al. 2006) and most vent macrofauna species reestablish within 5 yr (Shank et al. 1998). Recent eruptions occurred in 1991 and 2006, and our AST sample collections were performed between 1999 and 2004, during a period with very little change in meiofaunal or macrofaunal community structures. In addition, MDS plots and group-average linkage (PRIMER v. 5) showed sample clustering by habitats rather than year of sampling (see Fig. 4 in Gollner et al. 2010 for meiofauna, and Fig. S1 in our Supplement for macrofauna).

Observed species richness (*S*_obs_) and Shannon diversity were calculated from quantitative species-abundance data by the DIVERSE subroutine in PRIMER v. 5 (Clarke & Gorley 2001). Size-based rarefaction and extrapolation of species richness after identifying 300 ind. (*S*_m300_) and of species richness at a sample coverage of 98% (*S*_Cm0.98_) was obtained via iNEXT (Chao & Jost 2012, Hsieh et al. 2013). Significant differences of univariate measures were tested on transformed data using bootstrapping (10 000 resamplings each, 2-sided *t*-test, routine ‘FTBOOT’ from the package ‘computer intensive statistics’, available at http://homepage.univie.ac.at/hans.leo.nemeschkal) (Nemeschkal 1999). In addition to the FTBOOT routine (a program developed for studies with low sample size and high variances), we also applied non-parametric Kruskal-Wallis tests, with post hoc multiple comparisons (Mann-Whitney *U*-tests, 2-tailed with Bonferroni adjustment), using the program STATISTICA. Here we present results of the FTBOOT routine and results of both tests are shown in Table S3. The occurrence of species (presence/absence) in the different habitats was used to evaluate the meiofaunal and macrofaunal habitat fidelity. We distinguish between habitat specialists (species that occur in just a single habitat, i.e. Pompeii worm, tubeworm, mussel, or proximate basalt habitat) and habitat generalists (species that occur in at least 2 different habitats). To get a complete picture of species occurrence, we combined our species presence/absence data from different habitats with previously published meiofaunal occurrence data (Gollner et al. 2010 and citations therein) and macrofaunal occurrence data (Micheli et al. 2002, Van Dover 2003, Govenar et al. 2005, Desbruyères et al. 2006, Govenar & Fisher 2007, Mills et al. 2007b, Galkin & Goroslavskaya 2008, Matabos et al. 2008).

## RESULTS

Our sample analyses from within the AST comprised a total of ~175 000 ind. from 143 species (Table 2), apportioned as 61% meiofaunal and 39% macrofaunal species. Total species richness of meioand macrofaunal communities were both lowest in the Pompeii worm (11 meiofaunal species, 8 macrofaunal species) and intermediate in tubeworm (31 meiofauna, 35 macrofauna) and mussel habitats (36 meiofauna, 32 macrofauna). Proximate basalt exhibited highest meiofaunal species richness (total 64 species), but low macrofauna species richness (total 23 species).

**Fig. 1:**
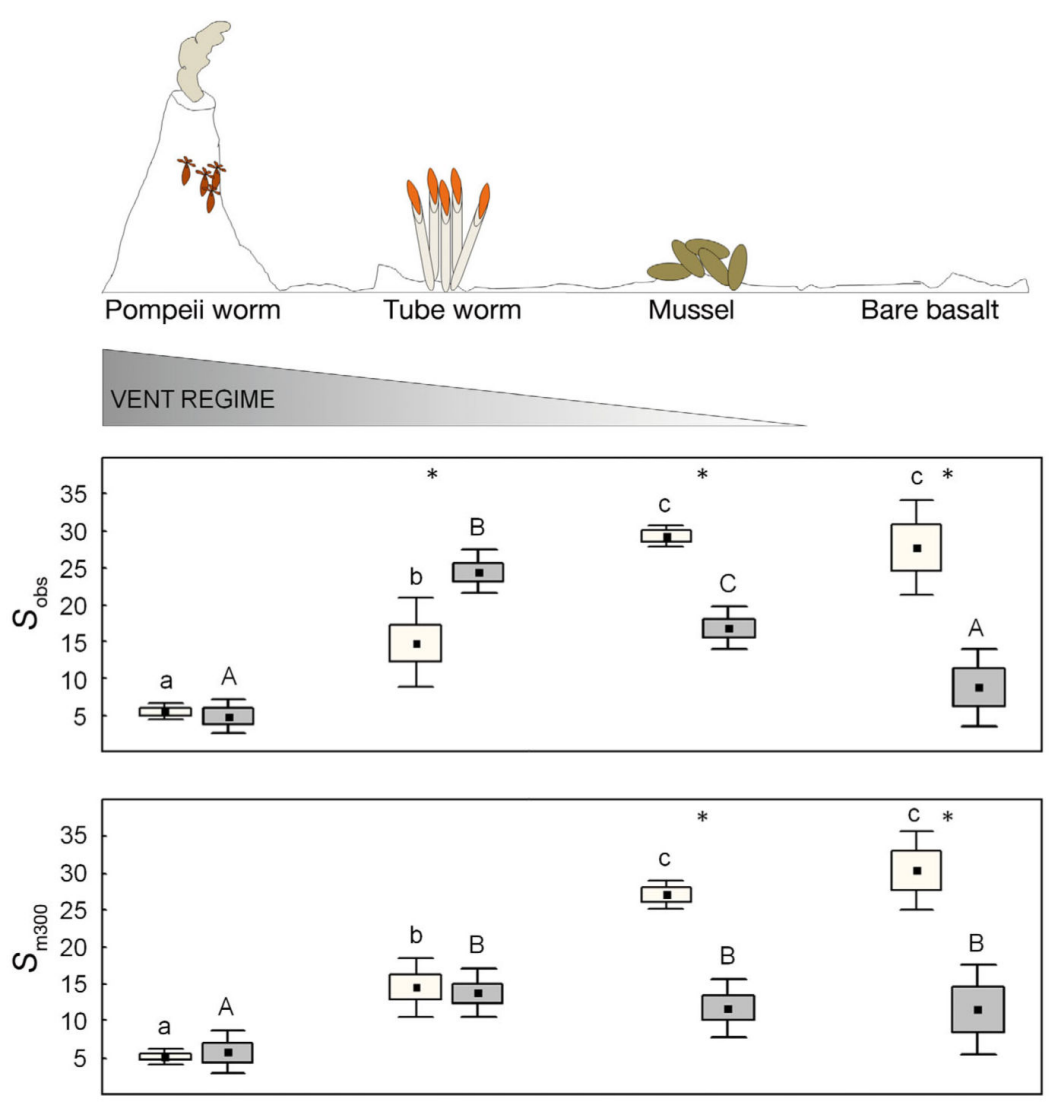
A schematic of the habitat types sampled, and the gradient of high temperatures and more concentrated vent fluids (‘vent regime’) from the Pompeii worm habitat to bare basalt. Box-and-whisker plots show observed species richness (*S*_obs_) and species richness at a sample size of 300 ind. (*S*_m300_) for meiofauna (white boxes) and macrofauna (grey boxes). Black square: mean; box: SE; whiskers: SD. Significant differences (p < 0.05) between meiofauna and macrofauna within a habitat are indicated by *. Letters shared in common between habitats indicate no significant differences for meiofauna (lowercase letters) and macrofauna (uppercase letters) separately

To account for differences in sampling methods, we also standardized species richness to sample area and sampling effort (Chao & Jost 2012) to compare across habitats. Species richness (*S*_obs_, *S*_m300_, *S*_Cm0.98_) and Shannon diversity for meiofauna increased significantly from Pompeii worm habitats (e.g. mean [±SD] *S*_m300_: 5 ± 1), to tubeworm (*S*_m300_: 14 ± 4) and mussel habitats (*S*_m300_: 27 ± 2). *S*_Cm0.98_ and the total number of species collected was highest from the proximate basalt (*S*_tot_: 64; *S*_Cm0.98_: 43 ± 16), but *S*_obs_, *S*_m300_, and Shannon diversity were similar between the mussel and the basalt habitats. Macrofaunal species richness (*S*_obs_, *S*_m300_, *S*_Cm0.98_) and Shannon diversity index were significantly lower at the Pompeii worm habitat (e.g. *S*_m300_: 6 ± 3) than at the tubeworm (*S*_m300_: 14 ± 3) and mussel (*S*_m300_: 12 ± 4) habitats. In contrast to the proximate basalt meiofauna, macrofaunal richness was low but variable (*S*_m300_: 11 ± 6) with ranges similar to Pompeii worm, tubeworm and mussel habitats. Interestingly, when considering only macrofauna >1 mm (i.e. adults), observed species richness values on basalt were extremely low (mean *S*_obs_: 2) (Fig. 1, Tables 2 & S3).

Of the 87 meiofaunal species found in the AST, 35 occurred exclusively on basalt, 29 co-occurred at vents and on basalt, and 23 occurred only at vents (see Gollner et al. 2010). In contrast, of the 56 macrofaunal species collected in the AST, 29 occurred only at vents, and 5 species occurred exclusively on basalt. Al though 22 macrofaunal species occurred at both vents and basalt, most individuals on basalt were juvenile stages. The vent-distant samples in cluded a total of 42 meiofaunal and 14 macrofaunal species. While 41% of the vent-distant meiofaunal species also occurred within the AST (Table S2), only 2 macrofaunal species, *Ophryothrocha akessoni* and a bentho-pelagic appendicularian, are known to occur within the AST (Desbruyères et al. 2006, S. Gollner pers. obs.).

Juvenile and adult meiofauna were found in all AST habitats (mean juveniles: 5–16%; Table 2). In contrast, adult macrofauna dominated hydrothermal habitats (mean juveniles: 0–8%) but juvenile macrofauna dominanted basalt (mean juveniles: 69%; Table 2). On proximate basalt, only 7 of the total 23 macrofauna species were larger than 1 mm (*Lepetodrilus elevatus*, *L. ovalis*, *L. cristatus*, *Rhynchopelta concentrica*, *Galapagomystides aristata*, *Ventiella sulfuris*, and *Dahlella caldariensis*).

## DISCUSSION

### Diversity and animal size

Diversity patterns along the environmental stress gradient differed remarkably for hydrothermal vent meio- and macrofaunal communities at the EPR. For meiofaunal communities, there was an increase in species richness from high- to low-stress habitats; for macrofaunal communities, species richness peaked in the intermediate stress habitats. We propose that life history traits and physiological capabilities inherent to size explain these differences. The small size of meiofauna results in small thermal mass, thin barriers to diffusion, and limited anatomical, physiological or behavioral options to deal with temperature fluctuations and chemical stress, and might be therefore more vulnerable than macrofauna to these forms of environmental stress (Powell, 1989, Brown et al. 2004). In contrast, many macrofaunal groups have evolved complex behavioral or physiological adaptations to the more extreme conditions in vent habitats (Childress & Fisher 1992, Rinke & Lee 2009, Bates et al. 2010).

The extreme spatial and temporal variation in temperature and chemical concentrations in the high stress Pompeii worm habitat correlated with low species richness for both meio- and macrofauna. The lower diversity appears to be a consequence of fewer species with adaptations to this extreme environment. No known metazoans can tolerate sustained body temperatures above 60°C (Lee 2003), and thus animals may avoid high temperatures by moving quickly (Shillito et al. 2001) or actively seeking cooler temperatures (Bates et al. 2010). Among the meiofauna, only a few copepod species that are known to move quickly (e.g. several cm s^−1^; S. Gollner & M. Bright, pers. obs.) were found in the Pompeii worm habitat, and we observed no slow-moving nematodes (a few mm s^−1^; S. Gollner & M. Bright, pers. obs.). The 2 most abundant macrofaunal species were the fast-swimming amphipod *Ventiella sulfuris* and the polychaete *Hesiolyra bergi*, which moves via rapid undulating movements (M. Bright pers. obs.). Similar dominance and richness patterns were also reported by Galkin & Goroslavskaya (2008) and by Pradillon et al. (2009) from sites with similar temperature ranges.

For both meio- and macrofaunal communities, species richness was greater in the intermediate stress habitats than in the Pompeii worm habitat. The less extreme conditions in tubeworm and mussel habitats may contribute to coexistence of species that can escape quickly and actively choose a suitable microhabitat in addition to species that can tolerate variable and extreme environmental conditions. Alternatively, the high species diversity in the intermediate stress habitats could be due to the complex 3-dimensional structure of the tubeworms and mussels (Govenar & Fisher 2007, Govenar 2010). The physical architecture of foundation species can increase surface area, habitat complexity, concentrate resources and larvae, and provide refuge from predation, which further facilitates species coexistence and thus enhances diversity (Bruno & Bertness 2001, Govenar & Fisher 2007). While macrofaunal diversity was greatest among tubeworm aggregations and increased with tube surface area (Govenar et al. 2005, Govenar & Fisher 2007), meiofaunal diver sity did not increase with tubeworm surface area (Gollner et al. 2007), and was greater among mussel aggregations which have lower shell surface area and less environmental heterogeneity. Two factors could contribute to these patterns. The gastropod grazers that dominate the macrofaunal community may benefit from the greater surface area of tubeworm tubes and also may be restricted from grazing among the interstices between byssal threads of the mussels, which would result in a refuge from predation that would selectively benefit the meiofauna.

Increased meiofaunal diversity with decreasing environmental stress suggests that meiofauna are more vulnerable than macrofauna to hydrothermal fluids, as they have very few physiological or ana tomical features to deal with chemical and thermal challenges. Given their small mass and large surface-area-to-volume ratio, temperature changes and toxic chemical compounds immediately affect homeostasis (Townsend & Thompson 2007). In addition, meiofauna are unable to develop effective physical protection such as thick shells or carapaces that would buffer against stress factors (e.g. temperature). In contrast to meiofauna, macrofauna have greater control over their physiological homeostasis and hence tolerate larger environmental variations (Brown & Sibly 2006). Several vent macrofauna species have developed energetically costly physiological adaptations, such as the production of heat-shock proteins (Ravaux 2003) or metabolic depression (Boutet et al. 2009), to deal with hydrothermal stress. Very abundant semi-sessile limpets can actively choose their hydrothermal environment (Bates et al. 2005) and can seal themselves from the environment using their thick shells, by periodically adhering tightly to the substrate like their intertidal relatives on rocky shores (Smith 1991). Thus, more diverse physiological options, potential for thick and robust protective coverings, and more diverse lifestyles in the form of mobile, semi-sessile, and sessile species of macrofauna give rise to high diversity in moderately stressful habitats.

On the low-stress basalt, meiofaunal richness was high but macrofaunal richness was low and dominated by juveniles. In the absence of environmental stress resulting from hydrothermal flow (see Table 1), other factors must contribute to low macrofaunal diversity. The lack of physical structures resulting from the absence of foundation species cannot explain low macrofaunal diversity on basalt, because the placement of artificial plastic tubes mimicking tubeworms on basalt did not enhance macrofauna diversity (Govenar & Fisher 2007). Instead, productivity and therefore food availability, which is much lower on the bare basalt than at vents (Etter & Mullineaux 2001, Govenar & Fisher 2007), is a likely cause. The dominance of juvenile macrofaunal species on the basalt suggests that these animals might obtain enough food while small but need to migrate into more productive, nutrient-rich vent habitats to develop into adults and reproduce. Similarly, Marcus & Tunnicliffe (2002) observed a decreasing limpet body size with distance from vents on the Juan de Fuca Ridge. Thus, the limited productivity on basalt in the AST may account for the relatively low macrofaunal diversity compared to more productive, albeit more stressful vent environments. For the meiofauna, we suggest that the low productivity on basalt is still sufficient to satisfy the energetic needs of relatively diverse communities.

In addition to the influence of hydrothermal stress, habitat complexity, and food availability, predation could have contributed to the observed abundance and diversity patterns. Our data also support the increase in negative species interactions along a gradient of increasing environmental stress, proposed by Mullineaux et al. (2003). Contrary to the expectations of the cross-community scaling relationship, in which body size and abundance are inversely related (White et al. 2007), macrofaunal abundance was not greater than meiofaunal abundance in tubeworm aggregations, suggesting predation by macrofauna on meiofauna. In the comparatively more benign environments of mussel aggregations, meiofaunal abundance was greater than macrofaunal abundance, indicating that the effect of predation may have been less where environmental stress was also less. Macrofaunal abundance and richness were low at the high-stress Pompeii worm habitat as well as the low-stress and food-poor basalt habitat, suggesting little influence of predation by macrofauna on meiofauna on these results, but abiotic controls instead.

### Distribution, habitat fidelity, and animal size

In contrast to the vent-restricted macrofauna, most meiofaunal species in areas with hydrothermal flow in the 9° 50′ N EPR region also occurred on basalt, suggesting a broad realized ecological niche and wide distribution. We propose 3 factors that might explain why so few meiofaunal vent specialists have arisen over evolutionary time. First, the higher food availability at vents may not provide enough of an energetic advantage to drive the evolution of adaptations to the hostile vent environment, because alternative food sources available outside of areas ex posed to hydrothermal flow are sufficient for their small body size. This interpretation follows the hypo thesis Mironov et al. (2001) developed for shallow-water vents, although neither the macrofauna nor the meiofauna at those shallow-water vents are restricted to vent habitat. They argued that there is no energetic advantage in developing complex adaptations to the toxic vent environment in shallow waters, given the abundant alternative food sources in the photic zone (Mironov et al. 2001, Tarasov et al. 2005). Second, meiofauna at vents might experience strong competition and predation pressure from the abundant macrofauna. Third, environmental stress associated with the more productive vent habitats may exclude meiofauna simply because their small mass and lack of thick protective structures prevent maintenance of physiological homeostasis in harsh and fluctuating vent habitats (see discussion above).

One higher meiofaunal taxon violates the pattern of generalist meiofauna: dirivultid copepods can be very abundant at vents and rarely occur on basalt. In addition, they have evolved a highly specialized diet, feeding on chemoautotrophic bacteria from vents (Limen et al. 2008), can potentially escape predation pressure by fast movement (see discussion above), and have evolved adaptations to the extreme hydrothermal regimes including high swimming speeds and the presence of hemoglobin with a very high oxygen affinity (Hourdez et al. 2000). Furthermore, dirivultid copepods at vents are much larger in volume and biomass than most meiofauna. In tubeworm aggregations, dirivultid copepods were, on average, 1.5 mm long and 0.12 mg in mass, while the much thinner harpacticoid copepods were about 0.8 mm in length and only 0.01 mg, and nematodes were about 0.7 mm long and only 0.0002 mg (data from Gollner et al. 2006, 2007). Thus, within the meiofauna, size correlates with evolutionary traits leading to vent habitat specialization.

To conclude, different food requirements of meioand macrofauna are important driving factors for the differences in the distribution and habitat fidelity patterns between meio- and macrofauna in the deep-sea. Life history traits and physiological capabilities inherent to size further refine these distribution patterns, leading to different patterns of species diversity and abundance among meio- and macrofaunal communities along an environmental stress gradient, from seafloor basalt to the Pompeii worm vent habitat. We propose that body size and related energy demands for reproduction are main drivers in the evolution of specialized vent fauna. We also propose that this specialization is not due to limited recruitment to vent habitats, but instead, the specialized macrofauna are food-limited on the bare basalt and must migrate into the vents to develop into adults and reproduce. In contrast, the majority of meiofaunal species appear to be physiologically able to live, feed and reproduce both at vents and on proximate and distant basalt.

## Supplementary Material

1

## Figures and Tables

**Table 1:** Environmental characteristics and faunal collections for Pompeii worm, tubeworm, mussel habitats, and proximate and distant basalt habitats, associated with deep-sea hydrothermal vents. Productivity categories are expressed as high or low according to presence/absence and distance to *in situ* primary production. Stress levels were measured by maximal values of temperature, pH and sulfide categories, as well as by changes in and gradients of temperature.

	Pompeii worm	Tubeworm	Mussel	Proximate basalt	Distant basalt
**Environmental characteristics**					
*In situ* primary production	Present	Present	Present	Absent	Absent
Productivity	High	High	High	Low	(Very) low
Stress due to hydrothermal activity	High	High–intermediate	Intermediate–low	Absent	Absent
Foundation species at studied sites	*Alvinella pompejana*	*Riftia pachyptila*	*Bathymodiolus thermophilus*	None	None
Max. temp. (°C) at sites of faunal collection	14–119	32–54	10	2	2
Temp. fluctuations within a few seconds (°C)	40*	5 to 15	5	0	0
Temperature gradient among foundation species	14–80*	40–50	~8	–	–
Max. sulfide (μM Σ H_2_S)	1520*	85–283	151	0	–
Min. pH	4*	4.4–5.7	6.7	8.1	–
Chemical data source	Le Bris & Gaill (2007)	Le Bris et al. (2006)	Le Bris et al. (2006)	Measured by N. Le Bris	
**Faunal collections**					
Sites	M-Vent, Michel’s Vent, Alvinella Pillar, Bio 9	Tica, Riftia Field	Eastwall (mussel bed)	Near Tica (~10 m), near Alvinella Pillar	~1 km west of Tica, outside the AST
Collection type	Grab	Bushmaster Jr.	Scoop, mussel pot	Grab	Grab, sediment slurp
Same faunal collection meio & macro	Yes	Yes	No	Yes	Yes
Year of collection	2004	2001 & 2002	2002 meio; 1999 macro	2003 & 2004	2006
Number of samples	5 meio & 5 macro	6 meio & 6 macro	3 meio & 6 macro	4 meio & 4 macro	3 meio & 3 macro
Meiofaunal data source	Gollner et al. (2010)	Gollner et al. (2010)	Gollner et al. (2010)	Gollner et al. (2010)	This study
Macrofaunal data source	This study	Govenar et al. (2005)	Van Dover (2003)	This study	This study

Values marked with ^*^ were obtained at comparable Pompeii worm sites by Di Meo-Savoie et al. (2004) and Le Bris & Gaill (2007). (–) not applicable or not measured

**Table 2:** Number of samples (N), total sampled area, total faunal abundance, and mean (±SD) abundance per 10 cm^2^, percentage of juveniles, species richness (*S*_tot_: total species richness; *S*_obs_: observed species richness; ${\hat{C}}_{S_{\mathrm{obs}}}$: mean percent sample coverage; *S*_m300_: species richness at a sample size of 300 ind.; *S*_Cm0.98_: species richness at a sample coverage of 98%) and Shannon diversity index (*H*′_loge_) for meiofauna and macrofauna within each habitat type. na: not applicable

Habitat	N	Total sampled area (cm^2^)	Abundance		%	*S*_tot_	*S*_obs_	${\hat{C}}_{S_{\mathrm{obs}}}$	*S*_m300_	*S*_Cm0.98_	*H* ′ _loge_
			Total	10 cm^−2^	juveniles						
**Meiofauna**											
Pompeii worm	5	565	10394	213 ± 175	0.3 ± 0.5	11	5 ± 1	100 ± 0	5 ± 1	3 ± 1	0.3 ± 0.1
Tubeworm	6	4300	35842	178 ± 391	6 ± 6	31	14 ± 6	98 ± 2	14 ± 4	13 ± 4	1.4 ± 0.6
Mussel	3	2770	20882	72 ± 15	16 ± 7	36	29 ± 2	100 ± 0	27 ± 2	27 ± 2	2.5 ± 0.1
Basalt	4	1356	2654	18 ± 23	5 ± 3	64	28 ± 6	86 ± 16	30 ± 5	43 ± 16	1.9 ± 0.7
**Macrofauna**											
Pompeii worm	5	565	188	4 ± 1	0 ± 0	8	5 ± 2	90 ± 12	6 ± 3	5 ± 2	1.1 ± 0.4
Tubeworm	6	4300	95753	278 ± 263	8 ± 10	35	25 ± 3	100 ± 0	14 ± 3	12 ± 3	1.8 ± 0.4
Mussel	6	3186	9155	29 ± 24	na	32	17 ± 3	99 ± 1	12 ± 4	11 ± 5	1.4 ± 0.3
Basalt	4	1356	477	3 ± 4	69 ± 37	23 (6)^a^	9 ± 5 (2 ± 3)^a^	89 ± 15	11 ± 6	14 ± 14	1.3 ± 0.2

a *S*_tot_ and *S*_obs_ when only accounting for macrofauna >1 mm. Exclusion of juvenile macrofauna only diminished macrofaunal species richness in basalt habitat collections
