# Supplementary material for: Size matters at deep-sea hydrothermal vents: different diversity and habitat fidelity patterns of meio- and macrofauna
Source: Mar Ecol Prog Ser. Author manuscript; Available in PMC 2015 Jul 9. (PMC4496463; doi:10.3354/meps11078)
Supplement: 1 [file NIHMS62333-supplement-1.pdf]

The following supplement accompanies the article

Size matters at deep-sea hydrothermal vents: different diversity and habitat fidelity patterns of meio- and macrofauna

Sabine Gollner*, Breea Govenar, Charles R. Fisher, Monika Bright

*Corresponding author: sabine.gollner@senckenberg.de

Marine Ecology Progress Series 520: 57–66 (2015)

Supplement

Table S1. Macrofaunal species abundance per 10 cm^2 is shown for each sample from the Pompeii worm habitat (P1 – P5) and the basalt habitat (B1 – B4). For each species the taxon is given (AN = Annelida, AR = Arthropoda, CH = Chordata, MO = Mollusca). **Bold** numbers indicate abundance > 1 individual per 10 cm^2

Species	Taxon	P1	P2	P3	P4	P5	B1	B2	B3	B4
Alvinella caudata	AN	**1.06**	0.35	**3.37**	**2.54**	**1.21**				
Alvinella pompejana	AN	**3.1**	**1.31**	**2.48**	**6.76**	**3.03**				
Amphisamytha galapagensis	AN	0.09	0.06				0.53	0.68		0.05
Archinome rosacea	AN						0.2	0.27		
Branchinotogluma sp. 1	AN									0.22
Flabelliderma sp. 1	AN						0.02		0.04	0.03
Galapagomystides aristata	AN									0.05
Glycera tesselata	AN							0.15		
Hesiolyra bergi	AN	0.97	0.61	0.89	**3.38**	0.61				
Hesiospina vestimentifera	AN									0.05
Juvenile polychaete	AN									0.03
Juvenile polynoid polychaete	AN							0.04		
Lepidonotopodium williamse	AN									0.03
Nectochaeta larvae	AN			0.18						
Nereis sandersi	AN						0.13	0.11		
Nicomache arwidsoni	AN							0.11		
Ophryotrocha akessoni	AN	0.18	0.12	0.18		0.61	0.02			0.73
Paralvinella grasslei	AN	0.35	0.41	0.35	2.54					0.05
Syllidae sp. 1	AN						0.02			
Amphipod sp. 4	AR						0.02	0.04		
Bythograea thermydron	AR		0.06							
Dahlella caldariensis	AR									0.22
Typhlotanais sp.1	AR						0.02			0.22
Ventiella sulfuris	AR	**1.06**	**2.13**	0.18		**2.42**	0.07			**6.88**
Tunicate	CH								0.04	
Gorgoleptis spiralis	MO									0.03
Juvenile gastropod	MO						0.15	0.19		
Juvenile limpet	MO						0.11	0.23		0.11
Lepetodrilus ovalis	MO									0.11
Lepetodrilus cristatus	MO									0.03
Lepetodrilus elevatus	MO		0.03	0.35					0.19	0.81
Lepetodrilus galriftensis	MO		0.03	0.18						
Rhynchopelta concentrica	MO								0.04	0.16

Table S2. Total abundance, species richness and species abundance of meiofauna and macrofauna from vent-distant samples collected ~1 km away from the AST axis at the 9°N EPR. Meiofauna and macrofauna (meio, macro) species are given according to higher taxa (AN = Annelida, AR = Arthropoda, CH = Chordata, MO = Mollusca, COP = Copepoda, NEMA = Nematoda, OSTRA = Ostracoda) for vent-distant sample B1 (v-d B1), and vent-distant samples S1 (v-d S1) and S2 (v-d S2). Shared species with the AST at vents and bare basalt are indicated by x. x* species were not included in Gollner et al. (2010; PLoS ONE 5(8): e12321, doi:10.1371/journal.pone.0012321) (bentho-pelagic species), but were observed by S. Gollner. Calanoida, Corycaeidae, Oncaeidae are here counted as 1 species each. In brackets: shared species including yet unpublished information from samples collected by S. Gollner and M. Bright in the AST after 2006 (unpublished shared species in the AST include *Barathricola rimensis*, *Ectinosoma* sp. 3, *Sarsameira* sp. 2, *Tisbe* sp. nov. 1, *Tisbe* sp. 2, Nematoda sp. 2)

	Taxon	Size	v-d B1	v-d S1	v-d S2	AST vent	AST basalt
**Abundance**		Macro	53	0	6		
		Meio	101	20	55		
**Species richness**		Macro	9	0	5		
		Meio	13	8	32		
**Species**							
Ophryotrocha akessoni	AN	Macro	1	0	0	x	x
Polychaeta off-axis sp. 1	AN	Macro	0	0	1		
Polynoid off-axis sp. 1	AN	Macro	1	0	0		
Amphipoda off-axis sp. 1	AR	Macro	1	0	0		
Amphipoda off-axis sp. 2 (juv)	AR	Macro	1	0	0		
Amphipoda off-axis sp. 3 (juv)	AR	Macro	1	0	0		
Cumacea	AR	Macro	0	0	2		
Isopoda off-axis sp. 1 (juv)	AR	Macro	0	0	1		
Isopoda off-axis sp. 2	AR	Macro	1	0	0		
Isopoda off-axis sp. 3	AR	Macro	0	0	1		
Isopoda off-axis sp. 4 (juv)	AR	Macro	0	0	1		
Tunicate (?)	CH	Macro	5	0	0		
Appendicularia	CH	Macro	41	0	0	x*	x*
Gastropoda off-axis sp. 1	MO	Macro	1	0	0		
Ameira sp. nov. 1	COP	Meio	1	0	2	x	x
Amphiascus sp. 1 (aff. varians)	COP	Meio	0	0	3	x	x
Aphotopontius acanthinus	COP	Meio	13	0	1	x	
Argestes angolaensis (?)	COP	Meio	0	0	1		
Barathricola rimensis	COP	Meio	0	1	1	(x)	(x)
Ectinosoma sp. 3	COP	Meio	0	0	1	(x)	
Idyella sp. 1	COP	Meio	0	0	2		
Idyella sp. 2	COP	Meio	0	0	1		
Keraia juvenile	COP	Meio	0	0	1		
Marsteinia sp. 1	COP	Meio	0	0	1		
Marsteinia sp. 2	COP	Meio	0	0	1		
Mesocletodes sp. nov. 2	COP	Meio	0	1	0		
Misophrioida sp. 1	COP	Meio	0	0	1		
Neobradia sp. 1	COP	Meio	0	0	1		
Paranannopidae juvenile	COP	Meio	0	0	1		
Pontostratiotes sp. 1	COP	Meio	0	1	0		
Pontostratiotes sp. 2	COP	Meio	1	0	0		
Pseudotachiidae sp. 1	COP	Meio	1	0	0		
Sapphirina sp. nov. 3	COP	Meio	1	0	0		
Sarsameira sp. 2	COP	Meio	0	0	2		(x)
Siphonostomatoida sp. 1	COP	Meio	0	0	1		
Siphonostomatoida sp. 2	COP	Meio	0	0	1		
Siphonostomatoida sp. 3	COP	Meio	0	0	1		
Stygiopontius hispidulus	COP	Meio	2	3	1	x	x
Tachidiopsis sp. 1	COP	Meio	0	0	1		
Tisbe sp. 2	COP	Meio	1	0	0	(x)	(x)

Tisbe sp. nov. 1	COP	Meio	2	5	13	(x)	(x)
Calanoida	COP	Meio	32	5	2	x*	x*
Corycaeidae	COP	Meio	10	0	0	x*	x*
Microsetella norwegica	COP	Meio	2	0	0	x*	x*
Oncaeidae	COP	Meio	34	2	2	x*	x*
Chromadorita (?) sp. 1	NEMA	Meio	0	0	1	x	x
COK (Chromadorida?) sp. 2	NEMA	Meio	0	0	1		
Epsilonematidae sp. 1	NEMA	Meio	0	0	2		
Epsilonematidae sp. 2	NEMA	Meio	0	0	2		
Microlaimus cyatholaimoides	NEMA	Meio	0	0	1		
Nematoda sp. 2	NEMA	Meio	0	0	2	(x)	
Nematoda sp. 3	NEMA	Meio	0	0	1		
Oncholaimus sp. 1	NEMA	Meio	1	0	0		
Paracyatholaimus sp. 4	NEMA	Meio	0	0	1		
Thalassomonhystera fisheri	NEMA	Meio	0	2	0	x	x
Xylocythere vanharteni	OSTRA	Meio	0	0	2	x	x

Table S3. Results of bootstrapping (bt, 10000 resamplings each) used to test for significant differences in abundance per 10 cm^2, observed species richness (S_{obs}), species richness after identifying 300 individuals (S_{m300}), species richness at a sample coverage of 98% ($S_{Cm0.98}$) and Shannon diversity (H'_{loge}) between the habitats P (Pompeii worm), T (tubeworm), M (mussel), and B (basalt) for (A) meiofauna and (B) macrofauna. (C) Bootstrapping results for meiofauna versus macrofauna in the four habitat types (P, T, M, B) tested for abundance per 10 cm^2, S_{obs}, S_{m300}, $S_{Cm0.98}$ and H'_{loge}. Higher (>) and lower (<) values of meiofauna (me) and macrofauna (ma) indices are indicated for each habitat. Significant results after classical Bonferroni-correction are marked in **bold**. In brackets: results of non-parametric Kruskal-Wallis tests

(A) Meiofauna

**Habitat**	**Abund. 10 cm^{-2}**	S_{obs}	S_{m300}	$S_{Cm0.98}$	H'_{loge}
P - T	0.29 (0.35)	**<0.001** (0.45)	**<0.001** (0.53)	**<0.001** (0.53)	**<0.001** (0.09)
T - M	0.81 (1)	**<0.001** (0.59)	**<0.001** (0.73)	**<0.001** (1)	**<0.001** (1)
M - B	**0.003** (0.49)	0.58 (1)	0.21 (1)	**<0.001** (1)	0.07 (1)
P - M	**<0.001** (1)	**<0.001 (0.01)**	**<0.001 (0.02)**	**<0.001** (0.06)	**<0.001 (0.04)**
P - B	**<0.001** (0.12)	**<0.001 (<0.01)**	**<0.001 (0.002)**	**<0.001 (<0.001)**	**<0.001** (0.17)
T - B	0.32 (1)	**<0.001** (0.53)	**<0.001** (0.25)	**<0.001** (0.12)	0.2 (1)

(B) Macrofauna

**Habitat**	**Abund. 10 cm^{-2}**	S_{obs}	S_{m300}	$S_{Cm0.98}$	H'_{loge}
P - T	**<0.001 (<0.01)**	**<0.001 (<0.01)**	**<0.001 (0.03)**	**<0.001 (0.07)**	**<0.001** (0.06)
T - M	**<0.001** (0.68)	**<0.001** (0.62)	0.28 (1)	0.95 (1)	0.06 (0.89)
M - B	**<0.001** (0.24)	**0.002** (0.99)	0.84 (1)	0.76 (1)	0.52 (1)
P - M	**<0.001** (0.50)	**<0.001** (0.14)	**<0.001** (0.19)	**0.006 (0.41)**	0.13 (1)
P - B	0.51 (1)	0.09 (1)	**0.003** (0.6)	0.024 (0.56)	0.29 (1)
T - B	**<0.001 (<0.01)**	**<0.001 (0.03)**	0.41 (1)	0.66 (1)	**0.005** (0.38)

(C) Meiofauna-Macrofauna

**Habitat**	**Abund. 10 cm^{-2}**		H'_{loge}	
P	**<0.001 (<0.01)**	me >	**<0.001 (<0.01)**	ma >
T	0.21 (0.08)		0.06 **(<0.01)**	ma >
M	**<0.001 (0.04)**	me >	**<0.001 (0.02)**	me >
B	0.08 (0.25)		0.06 **(0.02)**	me >

**Habitat**	S_{obs}		S_{m300}		$S_{Cm0.98}$	
P	0.47 (0.67)		0.85 (0.92)		0.07 (0.17)	
T	**<0.001 (0.01)**	ma >	0.71 (0.63)		0.51 (0.63)	
M	**<0.001 (0.02)**	me >	**<0.001 (0.02)**	me>	**0.01** (0.12)	me>
B	**<0.001 (0.02)**	me >	**<0.001 (0.02)**	me>	**<0.001 (0.02)**	me>

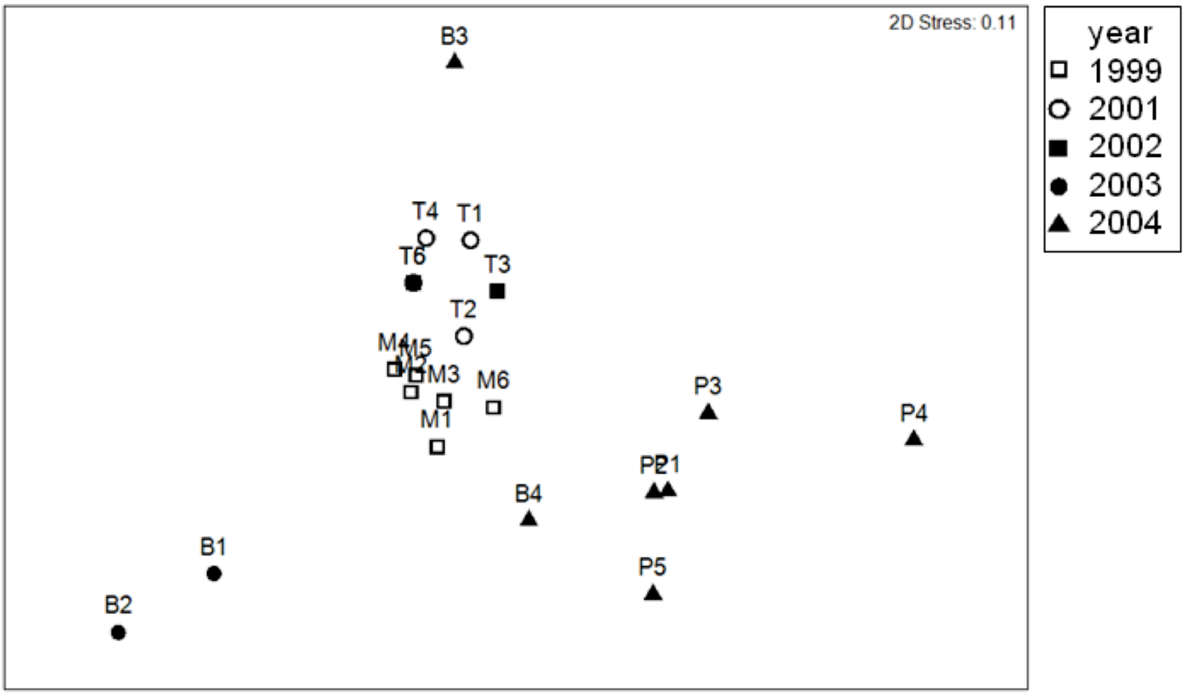

Figure S1. Two-dimensional MDS configuration plot (data standardized, square-root transformed; similarity based on Bray-Curtis similarity) of macrofauna data from Pompeii worm (P1-P5), tubeworm (T1-T6), mussel (M1-M6) and basalt habitats (B1-B4). Symbols indicate the year of sampling
